# 特别策划:庆祝《色谱》创刊40周年专辑

**DOI:** 10.3724/SP.J.1123.2024.05021

**Published:** 2024-06-08

**Authors:** Lihua ZHANG

## 引言

《色谱》作为广大分离分析学者心中的良师益友,转眼已步入不惑之年。一路相携走来,我们共同见证了《色谱》童年的欢乐、青年的憧憬和中年的奋进。在此,我谨代表色谱领域的广大学者和从业人员,向《色谱》致以最热烈的生日祝福,庆祝这四十载的光辉历程!

近年来,高效样品制备方法和分离材料、高峰容量分离模式、高灵敏度联用技术的不断发展,为色谱学科注入了强大的生命力,并已在自然科学、仪器制造、日常生活等方面占据了举足轻重的地位。它们不仅为生命科学前沿的复杂组学样品分析提供了高效、高分辨的分离手段,而且也在食品安全、环境检测、生物制药等领域为人民的生命健康保驾护航。同时,国产色谱仪器的日臻完善更是让我国在基础研究和应用开发中拥有了更多的话语权和自主权。

为庆祝《色谱》创刊40周年,我们诚挚邀请了色谱领域的知名学者和青年才俊,精心策划了由3篇视角、10篇专论与综述、9篇研究论文构成的“复杂体系高效分离与表征”和“组学分析新技术”两期专辑,内容涵盖样品制备、色谱固定相、联用技术、数据解析等多个方面,并深入探讨了这些技术在生命科学、生物医药、环境治理和食品安全等领域的最新研究进展。衷心希望这两期学术盛宴能够作为《色谱》40岁的生日贺礼,与广大读者共同举杯相庆。同时,我们也期待在未来的日子里,有更多的有识之士和青年才俊不断加入我们的队伍,共同为色谱事业的继往开来而努力奋斗。

在此,衷心感谢为“庆祝《色谱》创刊40周年专辑”撰写最新力作的每一位作者;衷心感谢为完善论文提出真知灼见的每一位审稿人;衷心感谢主编、副主编和编辑部老师的倾情奉献。谨以此专辑为《色谱》送上最真诚的生日祝福和最诚挚的感谢!

祝愿《色谱》的未来更加辉煌!

本专辑客座主编

中国科学院大连化学物理研究所

张丽华 研究员

2024年5月20日

